# First Documentation of Human Crimean-Congo Hemorrhagic Fever, Kenya

**DOI:** 10.3201/eid0809.010510

**Published:** 2002-09

**Authors:** Lee Dunster, Manuela Dunster, Victor Ofula, Dunston Beti, Femke Kazooba-Voskamp, Felicity Burt, Robert Swanepoel, Kevin M. DeCock

**Affiliations:** * World Health Organization Collaborating Centre for Arbovirus and Viral Hemorrhagic Fever Reference and Research, Nairobi, Kenya; † National Institute for Virology, Sandringham, Republic of South Africa; ‡ Centers for Disease Control and Prevention, Kenya Medical Research Institute, Nairobi, Kenya

**To the Editor:** On October 21, 2000, a previously healthy 25-year-old male farmer was admitted to a mission hospital in western Kenya with an acute hemorrhagic illness. Four days before admission, the patient had rapid onset of fever, headache, nausea, vomiting, severe muscle pains, and diarrhea, which became bloody. On admission his temperature was 36.4°C, pulse was 60/minute, respiratory rate was 20/minute, and blood pressure was 90/40 mm Hg. In addition to the signs and symptoms listed above, the only other abnormal finding on admission was neck stiffness. The differential diagnoses included bacterial dysentery and meningitis. Results of a blood smear for malaria parasites and Widal test for typhoid were negative, and cerebrospinal fluid and urine examinations were normal.

The patient was treated with doxycycline, cotrimoxazole, metronidazole, and intravenous fluids. On the day after admission, the patient’s vomitus became blood stained and blood was passed rectally. The patient was isolated and strict barrier nursing implemented on the suspicion of viral hemorrhagic fever (VHF). Progressive hypotension developed, resistant to resuscitation efforts with intravenous fluids and corticosteroids, and later massive bleeding from the nose, mouth, and upper and lower gastrointestinal tract occurred. The patient died on the second day of admission, 6 days after onset of illness. A serum sample was sent to the Arbovirus and Viral Hemorrhagic Fever Reference Laboratory in Nairobi for diagnostic screening.

Serologic tests in Nairobi were negative for yellow fever, dengue, West Nile, Chikungunya, and Rift Valley fever (immunoglobulin [Ig] M–capture enzyme-linked immunosorbent assay) and reverse transcriptase-polymerase chain reaction (RT-PCR) tests for flaviviruses, alphaviruses, and Bunyamwera serogroup bunyaviruses were also negative. RT-PCR for *Crimean-Congo hemorrhagic fever virus* (C-CHFV) was positive. Tests for anti-C-CHFV–specific IgM antibody by indirect immunofluorescence were negative. Virus isolation attempts were then terminated because the cultivation of C-CHFV (the presumptive cause) requires biosafety level 4 facilities. The specimen was submitted to the Special Pathogens Unit in Johannesburg for confirmation of the result. The sample was positive by RT-PCR for C-CHFV and was IgM and IgG antibody negative. No isolation of the virus could be made from the serum sample, possibly because it was received by the Johannesburg laboratory 8 days after initial collection and following freeze-thaw conditions. The specimen was was insufficient to attempt C-CHVF antigen detection assays. Sequencing of the RT-PCR amplicon confirmed C-CHFV.

C-CHFV is a tick-borne virus of the genus *Nairovirus*, family *Bunyaviridae,* and is widely distributed throughout eastern Europe and the Crimea, to the Middle East and western China, Pakistan, and Africa. Natural hosts for this virus are varied (including wild and domestic animals and birds) and may reflect the feeding preferences of the host tick (1). While C-CHFV infections are rare in humans, the virus is notorious for nosocomial outbreaks of VHF, typically following admission of an index case to a health-care facility where VHF was not suspected, with mortality rates up to 40%.

Previous evidence for C-CHFV in Kenya is limited and based on serology (human and bovine) and two isolations of C-CHFV from non-human sources (1,2). This report represents the first documented case of acute human C-CHFV infection in Kenya. The hospital concerned belongs to a VHF surveillance network serving to increase awareness and preparedness within Kenyan health-care facilities. In this case suspicion of VHF was raised, and the patient was immediately isolated, noninvasive procedures were instigated, and barrier nursing was implemented to prevent nosocomial transmission. No family or hospital staff member who had close contact with the patient became ill. Although VHFs are rare, this report stresses the need for health facilities in Kenya and East/Central Africa to include VHFs in their differential diagnosis of unexplained fever with hemorrhagic tendencies, as well as the utility of the surveillance network. The causative agents of Ebola hemorrhagic fever, Marburg hemorrhagic fever, C-CHFV, Rift Valley fever, and yellow fever are all endemic in East and Central Africa, and sporadic cases, as well as outbreaks, are likely to continue to occur in this region (3–5).

## References

[1] Hoogstraal H. The epidemiology of tick-borne Crimean-Congo hemorrhagic fever in Asia, Europe, and Africa. J Med Entomol. 1979;15:307–417. 113533 10.1093/jmedent/15.4.307

[2] Johnson BK, Ocheng D, Gichogo A, Okiro M, Libondo D, Tukei PM, Antibodies against haemorrhagic fever viruses in Kenya populations. Trans R Soc Trop Med Hyg. 1983;77:731–3. 10.1016/0035-9203(83)90216-X 6419422

[3] Rift Valley fever- East Africa 1997-1998. MMWR Morb Mortal Wkly Rep. 1998;47:261–4. 9565487

[4] Viral haemorrhagic fever/Marburg, Democratic Republic of the Congo. Wkly Epidemiol Rec. 1999;74:157–8. 10355355

[5] Outbreak of Ebola haemorrhagic fever, Uganda, August 2000-January 2001. Wkly Epidemiol Rec. 2001;76:41–6. 11233580

